# Dominant‐Negative Effects of p53 R337 Variants in Li–Fraumeni Syndrome: Impact on Tetramer Formation and Transcriptional Activity

**DOI:** 10.1002/cbic.202500330

**Published:** 2025-07-24

**Authors:** Rui Kamada, Shuya Sakaguchi, Madoka Kanno, Takaaki Ozawa, Natsumi Nakagawa, James G. Omichinski, Kazuyasu Sakaguchi

**Affiliations:** ^1^ Laboratory of Biological Chemistry Department of Chemistry Faculty of Science Hokkaido University Sapporo 060‐0810 Japan; ^2^ Département de Biochimie et Médicine Moléculaire Université de Montréal C.P. 6128 Succursale Centre‐Ville Montréal QC H3C 3J7 Canada

**Keywords:** DNA binding, dominant‐negative, hetero‐oligomerization, transcriptional activity, tumor suppressor protein p53

## Abstract

Li–Fraumeni syndrome (LFS) is an inherited cancer predisposition disorder caused by heterozygous *TP53* mutations. Among these, missense mutations at Arg337—such as R337C and R337H—are common in LFS patients. Although many studies have characterized individual p53 variants in LFS, the impact of tetramerization domain (TD) mutations on wild‐type (WT) p53 function remains unclear. Herein, a novel FRET‐based assay system that enables the simultaneous detection of heterotetramer formation and p53‐dependent transcriptional activity in live cells is developed. These results show that the heteromultimerization of the R337C variant with WT p53 is only slightly reduced compared to WT homotetramers, yet its transcriptional activity is diminished by over 50%. In contrast, the R337H variant forms heterotetramers at near‐normal levels but exhibits markedly compromised transcriptional activity. These findings reveal a previously unrecognized dominant‐negative‐like effect, suggesting reduced p53 function is due not only to decreased tetramer formation but also to diminished heterotetramer stability. Moreover, the LFS‐associated p53TD variants show a greater loss of activity against the low‐affinity, apoptosis‐inducing *bax* response element than against the high‐affinity, cell cycle arrest‐related *CDKN1A* response element. Collectively, this study demonstrates that p53TD mutations can exert dominant‐negative effects, advancing the understanding of p53 heteromultimer function in LFS pathogenesis. These mechanistic insights into p53 heterotetramer stability may not only inform genetic screening strategies for LFS but also support future therapeutic approaches aimed at restoring p53 function by stabilizing mutant tetramers.

## Graphical abstract

*Hetero‐tetramer dysfunction:* Li–Fraumeni syndrome (LFS) is a hereditary cancer predisposition caused by mutations in the *TP53* gene encoding the tumor suppressor p53. It is shown that p53 variants R337C and R337H, found in LFS, impair wild‐type (WT) p53 function by forming heterotetramers with reduced transcriptional activity. This dominant‐negative‐like effect may explain tissue‐specific tumor susceptibility in LFS.

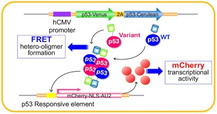

## 1 Introduction

Li–Fraumeni syndrome (LFS) is inherited genetic disorders,^[^ ^1^ ^]^ and patients with these disorders frequently develop juvenile cancers. The syndrome was first reported by Frederick Li and Joseph Fraumeni in 1969^[^ ^1a^ ^]^ and the syndrome is mainly caused by heterozygous mutations in the *TP53* gene.^[^ ^2^ ^]^ In most LFS patients, these mutations are present in a heterozygous state, meaning that the wild‐type (WT) and variant p53 are coexpressed.^[^ ^3^ ^]^ The *TP53* gene encodes for the tumor suppressor protein p53, which is a 393‐residue transcription factor that is activated in response to genotoxic stress and plays a central role in tumor suppression through transcriptional regulation of multiple target genes.^[^ ^4^ ^]^ To activate transcription, the p53 protein must adopt a homotetrameric structure, which requires a short tetramerization domain (TD) located between residues 326–356 in the sequence.^[^ ^5^ ^]^ Among the *TP53* mutations associated with LFS, missense mutations at Arg337 (for example, R337C and R337H) within the TD are particularly common.^[^ ^3^, ^6^ ^]^ The Arg residue at this position is highly conserved across vertebrate species and plays a critical role in stabilizing the structure of the p53 tetramer by forming a salt bridge with Asp352. Mutations that lead to substitutions that disrupt the formation of this key salt bridge often destabilize the tetrameric structure and impair p53 function. Notably, stabilization of the tetrameric structure has been reported to partially restore the function of R337H variants.^[^ ^7^ ^]^

Despite extensive studies on DNA‐binding domain mutations, the impact of TD mutations—especially under heterozygous conditions—remains poorly understood. In LFS patients, the co‐expression of WT and variant p53 raises important questions regarding how heterozygous expression of Arg337 variants affects overall tetramer formation and subsequent transcriptional activity. Arg337 plays an important role in the stability of the tetramer through the formation of a salt bridge with Asp354.^[^ ^8^ ^]^ Moreover, differential responses of various p53 target promoters (e.g., those driving cell cycle arrest versus apoptosis) may determine how these mutations modulate diverse cellular outcomes. Understanding the molecular basis of p53 dysfunction in LFS requires elucidating the effects of mutations on WT p53 function under heterozygous conditions.

Dominant negative effects can occur when variant proteins outcompete their wild‐type counterparts and inhibit normal protein function. In the case of p53, such effects arise when variant subunits containing mutated residues are incorporated into heterotetramers with wild‐type p53, resulting in complexes that are structurally intact but functionally impaired. These p53 heterotetramers often exhibit reduced DNA binding affinity and/or reduced transcriptional activation capacity, thereby suppressing the tumor suppressor function of the homotetrameric WT p53. Such effects are often attributed to amino acid substitutions in functional domains and are particularly common when they occur in multimeric proteins.^[^ ^9^ ^]^ Many *TP53* mutations in tumors are missense mutations, and they frequently occur within the DNA‐binding domain.^[^ ^10^ ^]^ In many cases, these mutations result in the formation of heterotetramers between WT and variant p53 that have the capacity to inactivate the protein through dominant negative effects. Although numerous studies have focused on individual p53 variants in LFS, the formation of heterotetramers between TD variants and WT p53 remains unclear. Therefore, analyzing hetero‐oligomer formation is critical to understanding the mechanisms of p53 inactivation and cellular oncogenesis in LFS, as any disruption in the ability of p53 to assemble a stable tetramer or to induce the necessary DNA bending will compromise its transcriptional activation function and overall tumor suppressor activity.

In this study, we have designed a novel assay system that allows for the simultaneous analysis of hetero‐oligomers formed by WT p53 and p53 TD variants, as well as their transcriptional activity for different p53‐dependent reporters in live cells. This system combines fluorescence resonance energy transfer (FRET)‐based detection with a transcriptional reporter, enabling quantitative and parallel measurement of oligomer formation and functional output within the same cellular context.

Using this approach, we determined that mutations at R337 of p53, which are associated with LFS (specifically R337C, R337H, and R337P), alter the stability of the heterotetramers formed with WT p53 as well as the transcriptional activity when co‐expressed in a heterozygous state. Our analyses demonstrated that these mutations exert a dominant‐negative or dominant‐negative‐like effect. Furthermore, differential responses at distinct p53‐dependent promoters suggest that these mutations have the capacity to modulate diverse cellular outcomes, including cell cycle arrest and apoptosis. These findings underscore the crucial role of heterotetramers formed between WT and variant p53 as a key component of the molecular basis of p53 dysfunction in LFS and lay the groundwork for a better understanding of LFS pathogenesis.

## 2 Results

### 2.1 Comparison of DNA Binding of p53 Proteins for *bax* and *CDKN1A* Response Elements

**Figure 1:**
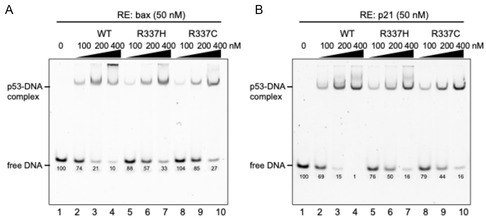
Gel shift assay of p53 proteins for A) *bax* and B) *CDKN1A*. A fixed concentration of Cy3‐labeled DNA probe (50 nM) with the indicated concentrations of p53 were incubated in 25 mM Tris‐HCl at pH9.2, 100 mM KCl, 1 mM MgCl_2_, 0.5 mM EDTA, 1 mM DTT, 0.1% NP‐40, 1 mg mL^−1^ BSA, 30% (v/v) glycerol for 1 h at 4 °C and were run on 4% nondenaturing acrylamide gels. Representative images of the gel shift assay were shown. Data are representative of two independent experiments. The percentage of free DNA was quantified by densitometric analysis of the Cy3‐labeled free DNA bands using image J software. The intensity of the free DNA band in the absence of protein was set to 100%, and the relative remaining intensity at each protein concentration was calculated accordingly. These values are shown beneath each band.

To analyze the DNA‐binding affinities of full‐length p53 proteins (WT, R337H, and R337C) at two promoter sequences, we first performed a gel shift assay. The p21 (*CDKN1A*) and *bax* promoters were selected because they are representative of p53 target genes involved in cell cycle arrest and apoptosis, respectively (**Figure** **1**). In addition to their distinct functional outcomes, these two genes are known to differ in their intrinsic binding affinity for p53, with *CDKN1A* generally exhibiting higher affinity than *bax*.^[^ ^11^ ^]^ This divergence allows us to evaluate whether p53 variants differentially impact transcriptional activation depending on promoter context and binding strength. Gel shift assays revealed that in the presence of either promoter, increasing the concentration of the p53 WT protein resulted in a concentration‐dependent increase in the amount of a p53‐DNA complex, which was quantitatively assessed by measuring the relative intensity of free DNA bands. Moreover, p53 bound to the p21 DNA with stronger affinity than to the bax DNA as expected. In the case of R337H p53, we observed weaker binding to both the p21 and bax DNA in comparison to the WT p53. In addition, the R337C p53 exhibited even lower affinity to both the p21 and bax DNA than did the R337H p53. These results suggest that the destabilization of the tetrameric structure of p53 by either the R337H or R337C variants leads to a decrease in the binding affinity of p53 to both the *CDKN1A* and *bax* promoters.

### 2.2 Analysis of Hetero‐Tetramerization by Avidin‐Biotin Pull‐Down Assay

**Figure 2:**
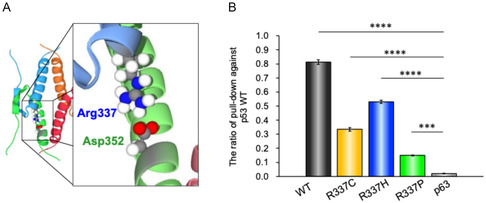
A) Structure of the p53 tetramerization domain. The structure is shown as a cartoon representation with each monomer depicted in a different color (cyan, green, orange, and red). Residues R337 and Asp352 are highlighted as ball‐and‐stick models. B) Ratio of variant p53TD pulled‐down versus WT p53TD. Experiments were performed in 50 mM Na phosphate buffer (pH 7.5), 100 mM NaCl, and peptide solutions containing the Bio‐(Aca)_2_‐p53TD and p53TD variants at equimolar concentrations (10 μM). The results demonstrate that the p53TD was pulled down by the WT(0.81), R337C (0.33), R337H (0.53), and R337P (0.15). No substantial pull‐down of p63TD was observed (0.02). Data represent the mean ± SD from three independent experiments. Statistical significance was assessed using Student's *t*‐tests comparing each variant to p63TD (negative control). ***p < 0.001, ****p < 0.0001.

Next, to examine whether the R337 variants of p53 form hetero‐tetramers with the WT p53, we first performed an avidin‐biotin pull‐down assay using peptides containing just the p53TD (residues 319–358 with and without the substitutions at R337) (**Figure** **2**A). In these experiments, a biotin‐labeled WT p53TD was mixed with nonbiotinylated variant p53TD peptides to determine whether it can form hetero‐oligomers with the R337 variants (**Table** **1**, Figure 2B). More specifically, we analyze the ratio of p53TD variant that is pulled down with respect to the amount of biotinylated p53TD present. The results indicate that each of the R337 variant p53TDs had the capacity to form heterotetramers with the WT p53TD in this in vitro assay. In these experiments, the WT‐to‐WT ratio was measured to be 0.81, whereas the ratio was 0.33 for the R337C mutation, 0.53 for the R337H mutation, and 0.15 for the R337P mutation when the WT and variant peptides were mixed at equal concentrations (10 μM). As a negative control for this experiment, we next measured the ability of the TD of p63 (residues 397–444 of p63) to form heterotetramers with the WT p53TD. These two TDs have been reported not to form heterotetramers, and as expected the pull‐down ratio for the p63TD with WT p53TD was only 0.02. The results demonstrate that the R337 mutations tested in this study (R337C, R337H, and R337P) can form heterotetramers with WT p53TD in vitro. These results strongly suggest that the R337 variants (R337H, R337C) associated with LFS have the potential to form heterotetramers with WT in cells when expressed as heterozygous mutations.

**Table 1:** The sequences of p53TDs with variations at R337 used in the pull‐down assay.

Variant name	p53 sequences
WT	KKKPLDGEYFTLQIRGRERFEMFRELNEALELKDAQAGKE
R337H	KKKPLDGEYFTLQIRGREHFEMFRELNEALELKDAQAGKE
R337C	KKKPLDGEYFTLQIRGRECFEMFRELNEALELKDAQAGKE
R337P	KKKPLDGEYFTLQIRGREPFEMFRELNEALELKDAQAGKE
	p63 Sequence
p63	DDELLYLPVRGRETYEMLLKIKESLELMQYLPQHTIETYRQQQQQQHQ

### 2.3 Construction of Dual‐Assay System for Hetero‐Tetramer Formation and Transcriptional Activity by FRET

In order to analyze hetero‐oligomerization between the WT p53 and LFS‐associated p53 TD variants in cells, a novel construct was created. In this construct, two p53 proteins are arranged in tandem. The first one is fused to the blue fluorescent protein Cerulean, the second is fused to the yellow fluorescent protein Venus, and there is a 2 A sequence of random amino acids between them. By using the construct, the WT‐p53 and the p53 TD variants (R337C and R337H) can be expressed in cells labeled with different fluorescent tags, which allows for the quantification of the amount of heteromeric p53 (WT with variants) formed based on the intensity of the FRET signal. In addition, a construct was also created to determine the transcriptional activity, which contains the red fluorescence protein mCherry with a nuclear localization signal that is under the control of a p53‐responsive element, *bax* promoter (Figure S1, Supporting Information).

**Figure 3:**
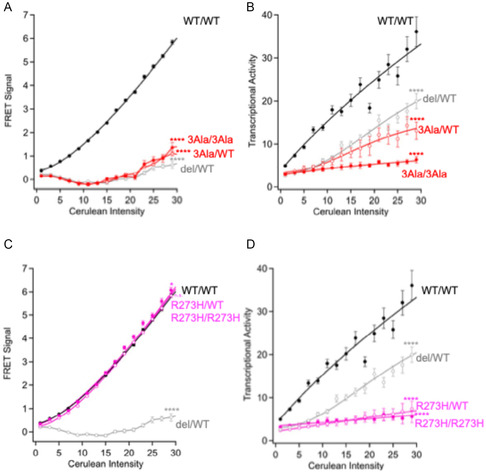
Analysis of p53 wild‐type, 3Ala, and R273H variant proteins using the FRET‐based Assay. Cells were transfected with phCMV‐p53(variant)‐Venus‐2A‐p53(WT)‐Cerulean (**Mut/WT**) and phCMV‐p53(variant)‐Venus‐2A‐p53(variant)‐Cerulean (**Mut/Mut**) and analyzed A,C) for formation of either homo‐ or hetero‐tetramers as well as B,D) for transcriptional activity with the pp53RE(*bax*)‐mCherry‐NLS‐AU2. A,B) 3Ala, C,D) R273H. **WT/WT** and **del/WT** p53 proteins were shown in black and gray, respectively. The FRET signals (oligomer formation) and the mCherry (p53‐dependent transcription) fluorescence signals in each single cell were quantified. Data represent the mean ± SEM from more than 1000 cells per condition, based on three independent experiments. Significance was analyzed using the Kruskal–Wallis test. *p < 0.05; ****p < 0.0001; n.s., not significant.

As a control for the system, we first examined homotetramer formation and transcriptional activity of the WT p53 protein using the pp53RE(*bax*)‐mCherry‐NLS‐AU2 and phCMV‐p53(WT)‐Venus‐2A‐p53(WT)‐Cerulean (**WT/WT**). To do this, p53(−/−) H1299 cells were cotransfected with the two plasmids, and both the FRET signal and mCherry signal were observed. Using this system, the FRET signal and mCherry signals were directly correlated to the p53 expression level, indicating that formation of the homotetramer and transcriptional activity can be detected in a p53‐dependent manner using this system (**Figure** **3**A,B and Figure S1, Supporting Information).

Next, the 3Ala variant of p53 (p53‐3Ala) was analyzed using the same FRET assay. In the p53‐3Ala protein, L330, L344, and I332 of p53 are substituted with an Ala residue, and these substitutions prevent p53 from forming multimeric structures (dimers or tetramers). Using the FRET‐based system, we failed to observe a signal with the p53‐3Ala variant following either homo‐ (phCMV‐p53(3Ala)‐Venus‐2A‐p53(3Ala)‐Cerulean; **3Ala/3Ala**) and hetero‐ (phCMV‐p53(3Ala)‐Venus‐2A‐p53(WT)‐Cerulean; **3Ala/WT**) expression with WT p53, which indicates that it fails to form multimeric structures with either itself or with WT p53. In addition, little to no transcriptional activity was observed with either the homo‐ and hetero‐expression of p53‐3Ala and this is similar to what is observed following transfection of a hCMV‐Venus‐NLS‐2A‐p53(WT)‐Cerulean plasmid, which serves as a model for the hetero‐deletion of *TP53*. Taken together, these results indicated that the p53‐3Ala variant does not have the capacity to form a heterotetramer with WT p53 and therefore does not alter its transcriptional activity (Figure 3A,B).

Next, the R273H variant of p53 was analyzed using the cellular FRET assay. The R273H mutation is located in the DNA‐binding domain (DBD) of the p53 protein. This variant has been previously shown to exert dominant‐negative effects on p53. In the FRET assay, the levels of tetramer formed with this variant were similar to what is observed from (**WT/WT**) p53 following either homo (phCMV‐p53(R273H)‐Venus‐2A‐p53(R273H)‐Cerulean; **R273H/R273H**) or hetero (phCMV‐p53(R273H)‐Venus‐2A‐p53(WT)‐Cerulean; **R273H/WT**) expression, which indicates that this mutation does not alter the ability of WT p53 to form tetramers. However, the (**R273H/R273H**) p53 protein displayed no transcriptional activity. Furthermore, very low transcriptional activity was observed following hetero‐expression of the (**R273H/WT**) p53 protein as the levels were even lower than those obtained following the expression of the heterozygous deletion (**del/WT**) protein. This indicates that a dominant‐negative effect was observed with the R273H variant, as heterologous expression greatly reduced the transcriptional activity of the WT p53 protein (Figure 3C,D).

These results demonstrate that this FRET system can be reliably used to quantify both the amount of hetero‐tetramerization and the transcriptional activity of p53 variants as well as determine whether they have a dominant‐negative effect on p53 activity.

### 2.4 p53 Tetramerization and Transcriptional Activity for Different Response Elements

Using the verified FRET‐based assay described above, we next analyzed the transcriptional activity of the R337 variants of p53 that have been associated with either the Li–Fraumeni syndrome or a Li–Fraumeni‐like syndrome. More specifically, the transcriptional activity of the R337C, R337H, and R337P p53 variant proteins were measured with the p53RE(*bax*) gene using the FRET system.

**Figure 4:**
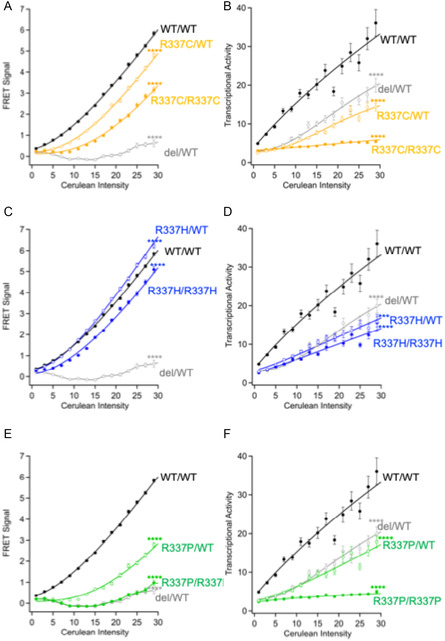
Analysis of p53 R337 variants using the FRET‐based assay. Cells were transfected with phCMV‐p53(variant)‐Venus‐2A‐p53(WT)‐Cerulean (**Mut/WT**) and phCMV‐p53(variant)‐Venus‐2A‐p53(variant)‐Cerulean (**Mut/Mut**) and analyzed A,C,E) for formation of either homo‐ or hetero‐tetramers as well as B,D,F) for transcriptional activity with the pp53RE(*bax*)‐mCherry‐NLS‐AU2. A,B) R337C, C,D) R337H, E,F) R337P. **WT/WT** and **del/WT** p53 proteins were shown in black and gray, respectively. The FRET signals (oligomer formation) and the mCherry (p53‐dependent transcription) fluorescence signals in each single cell were quantified. Data represent the mean ± SEM from more than 1000 cells per condition, based on at least three independent experiments. Significance was analyzed using the Kruskal–Wallis test. ****p < 0.0001.

Using this assay, we first measured the amount of homotetramerization observed with the R337C (**R337C/R337C**) variant of p53 and obtained a FRET signal of 0.7 at Cerulean Intensity 15 (**Figure** **4**A,B). Since the FRET signal of **WT/WT** at the equivalent point was measured at 2.3, the amount of homotetramerization of R337C variant is only about 30% of that observed with the WT p53. This result indicates that the amount of homotetramerization of R337C p53 is lower than the WT but still present in an appreciable amount. Interestingly, the R337C variant showed no transcriptional activity from *bax* promoter despite that fact that it still formed an appreciable amount of homotetramers. This level of transcriptional activity is similar to what is observed with the p53‐3Ala variant, which does not have the capacity to form multimeric structures (dimers or tetramers). However, the FRET signal measured for the heterotetramer formed with R337C and WT p53 (**R337C/WT**) was 1.6, which is about 70% of the amount for the WT homotetramer. Despite the fact that the R337C variant could form a considerable amount of heterotetramer with WT p53, the transcriptional activity of the **R337C/WT** was even lower than that of the heterozygous deletion model **del/WT**. Taken together, the results indicate that the R337C variant of p53, when heterologously expressed, has a dominant‐negative effect that reduces the transcriptional activity of the WT p53 protein.

In a similar manner, we evaluated the formation of multimers and the transcriptional activity of the R337H p53 variant using the FRET assay. The R337H variant formed homotetramers (**R337H/R337H**) and gave a FRET signal of 1.7, which was about 80% of **WT/WT** (Figure 4C,D). This value was higher than that observed with the R337C variant. However, the transcriptional activity of **R337H/R337H** from *bax* promoter was similar to that observed with the **del/WT**. When mixed with the WT p53, the amount of heterotetramer (**R337H/WT**) formed by the R337H variant was slightly higher than that observed for the WT homotetramer. However, the transcriptional activity of the **R337H/WT** heterotetramers was greatly reduced relative to the WT p53 as it was at roughly the same level as **del/WT**.

Lastly, we evaluated the formation of tetramers and the transcriptional activity of the R337P p53 variant using the FRET assay. Under the same experimental conditions, there was no FRET signal observed following expression of the R337 p53 variant, which suggests that there are no interactions (dimers or tetramers) between the R337P p53 monomers (Figure 4E,F). In contrast, hetero‐expression of the **R337P/WT** gave a FRET signal that was about 30% of that of **WT/WT** signal, which indicates that the R337P p53 has the capacity to form heterotetramers with the WT p53. In addition, the R337P variant was not transcriptionally active when expressed alone but showed the same activity as **del/WT** when coexpressed with the WT p53 protein, indicating that coexpression of R337P did not affect the transcriptional activity of WT p53.

To further investigate whether variations in protein affinity for DNA target sequences affect transcriptional activity, we examined the activity of R337 p53 variants associated with Li–Fraumeni syndrome or Li–Fraumeni‐like syndrome using a reporter driven by a *CDKN1A* response element (Figure S2, Supporting Information). This response element was selected because WT p53 exhibits a sixfold higher affinity for the *CDKN1A* responsive element in comparison with the *bax* responsive element.

As seen with the *bax* promoter, the **R273H/R273H** variant was not transcriptionally active with the *CDKN1A* response element under the experimental conditions (Figure S2A,B, Supporting Information). However, the transcriptional activity of the heterologously expressed **R273H/WT** was only slightly below the levels of the **del/WT**, and a significant dominant‐negative effect was not observed for this promoter as was seen with the *bax* promoter.

In contrast to what was observed with the *bax* response element, the R337C homo‐tetramer (**R337C/R337C**) displayed modest transcriptional activity with the *CDKN1A* promoter, while the heterozygous **R337C/WT** configuration produced activity comparable to that of **del/WT** (Figure S2C,D, Supporting Information). Similarly, the R337H variant induced measurable transcriptional activity from the *CDKN1A* promoter either when expressed as a homotetramer (**R337H/R337H**) or when coexpressed with WT p53 (**R337H/WT**). The levels of activity with the R337H variant were either similar to or higher than those of the **del/WT** model, but still lower than those of **WT/WT** (Figure S2E,F, Supporting Information). Finally, as observed with the *bax* promoter, the R337P variant failed to activate transcription in the homotetrameric form (**R337P/R337P**), whereas the heterozygous **R337P/WT** form exhibited transcriptional activity similar to **del/WT** (Figure S2G,H, Supporting Information).

### 2.5 Comparison of Transcriptional Activity for *bax* and *CDKN1A* Response Elements

**Figure 5:**
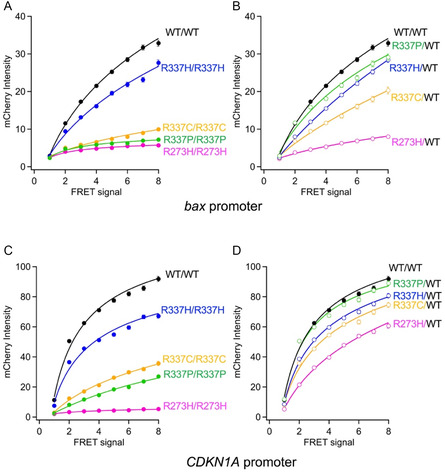
Summary of transcriptional activity for p53 variants. Data for *bax* promoter are from Figure 3 and 4, and data for *CDKN1A* promoter are from Figure S2, Supporting Information. Data represent the mean ± SEM from more than 1000 cells per condition, based on at least three independent experiments. This replot enables direct comparison of variant‐specific effects across two p53 target genes.

Since the ability of the R337 variants to form tetramers varied, their normalized transcriptional activity was analyzed in comparison with the levels of homotetramers formed with WT p53 (**WT/WT**). The results showed that only the R337H variant when coexpressed with WT p53 (**R337H/WT**) showed activity with the *bax* promoter (**Figure** **5**A,B). Furthermore, analysis of transcriptional activity with the *CDKN1A* promoter showed that heterologous expression of the R337H variant (**R337H/WT**) again displayed activity. More interestingly, **R337C/WT** and **R337P/WT** were found to be slightly active (Figure 5C,D).

To compare the transcriptional activity of each R337 variant against the *bax* promoter with that against *CDKN1A*, their transcriptional activity versus their ability to oligomerize was normalized with WT p53 (**WT/WT**) at Cerulean fluorescence intensities between 10 and 20 (Figure S3A, Supporting Information, **Table** **2**). The WT p53 (**WT/WT**) homo‐tetramer had ≈410% more activity with the *CDKN1A* promoter than with the *bax* promoter (Figure S3A, Supporting Information). The p53 heterozygous deletion model (**del/WT**) had 50% activity with the *bax* promoter and 230% activity with the *CDKN1A*, whereas the heterozygous R337 variant (**R337H/WT**) also had 50% activity with the *bax* promoter and 310% activity with the *CDKN1A*. The heterozygous R337P variant (**R337P/WT**) and R337C variant (**R337C/WT**) had similar transcriptional activity to the **del/WT** at 45% and 40% with the *bax* promoter and 250% and 240% with the *CDKN1A* promoter, respectively.

**Table 2:** Summary of tetramer formation analysis and transcriptional activity analysis in each variant.

Molecular functions	p53/p53	Mutantsa)				
		3Ala	R273H	R337C	R337H	R337P
Tetramerization	Mut/Mut	**‐**	**++**	**+**	**++**	**‐**
	Mut/WT	**‐**	**++**	**++**	**++**	**+**
Transcriptional activity (*bax*)	Mut/Mut	**‐‐**	**‐‐**	**‐‐**	**‐**	**‐‐**
	Mut/WT	**‐**	**‐‐**	**‐**	**‐**	**‐**
Transcriptional activity (*CDKN1A*)	Mut/Mut	**N.D.**	**‐‐**	**‐‐**	**‐**	**‐‐**
	Mut/WT	**N.D.**	**‐**	**‐**	**‐**	**‐**

a) The effect of homotetramer Mut/Mut and heterotetramer Mut/WT formation on transcriptional activity with the *bax* and *CDKN1A* promoters is summarized. If the activity of a particular tetramer was similar or identical to the activity of the del/WT tetramer, the result was categorized as “‐.” If the activity of a particular tetramer formation was similar to the activity of the WT/WT tetramer, the result was categorized as “++.” The activity of a tetramer was categorized as “+” if it was lower than that of the WT/WT tetramer, but displayed tetramer formation. If no transcriptional activity was observed, the result was categorized as “‐‐.” “N.D.” indicates that no data was obtained.

**Figure 6:**
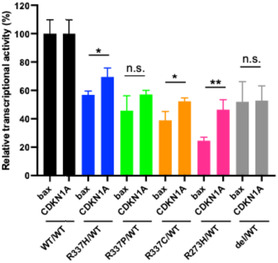
Relative transcriptional activity for each p53 variant at two target promoters. Transcriptional activity values for the *bax* promoter are from Figure 3 and 4, and those for the *CDKN1A* promoter are from Figure S2, Supporting Information. The transcriptional activity was normalized by WT/WT transcriptional activity against the *bax* response sequence and normalized by the respective WT/WT transcriptional activity against the *CDKN1A (p21)* response sequences. Data represent the mean ± SD from at least three independent experiments. Statistical significance was determined using Student's *t*‐test. *p < 0.05; **p < 0.01; n.s, not significant.

The transcriptional activity of each variant at the time of hetero‐tetramerization was normalized and analyzed with that of **WT/WT** for the *bax* promoter and *CDKN1A* promoter (**Figure** **6**). Differences in affinity between p53 and DNA were not taken into account. The DNA‐binding domain variant R273H showed 25% transcriptional activity against *bax* and 45% transcriptional activity against *CDKN1A*, both lower than **del/WT**, while R337C and R337P variants showed 40% and 45%, respectively, for the *bax* promoter and 50% and 55% for the *CDKN1A* promoter, respectively; these activities were comparable to **del/WT**, hetero deletion model of *TP53* gene. On the other hand, R337H showed a value of 55% for the *bax* promoter, similar to **del/WT**, and a slightly higher activity of 65% for the *CDKN1A* promoter.

## 3 Discussion

In this study, we successfully established a system for the simultaneous analysis of p53 expression level‐dependent tetramerization and transcriptional activation. The high correlation observed between tetramer formation and transcriptional output enabled us to evaluate the dominant‐negative and dominant‐negative‐like effects of R337 variants of p53 which have been associated with the Li–Fraumeni syndrome (LFS) and the Li–Fraumeni‐like syndrome.

Our analysis revealed distinct behaviors among the three R337 variants, which are found within the TD of p53. The R337C variant, which is frequently found in LFS patients,^[^ ^6d,6e^ ^]^ exhibited lower levels of homo‐tetramer formation, based on our FRET analysis experiments. The R337C homotetramer was severely compromised functionally as it displayed negligible transcriptional activity under our experimental conditions. Notably, when coexpressed with WT p53, the levels of heterotetramer formed with the R337C variant and WT p53 were slightly lower than the levels of homotetramer formation by the WT p53. This suggests that although the R337C variant p53 can form heterotetramers with the WT p53, the inability to form salt bridges with Asp352^[^ ^8^ ^]^ that are important for stabilization of the tetramer leads to less stable oligomeric structures with markedly reduced transcriptional function.

The R337C variant, which can only form homotetramers, showed low transcriptional activity, whereas the transcriptional activity of **R337C/WT**, when the R337C variant and WT p53 are coexpressed, was even lower than that of the **del/WT**, a heterozygous deletion model. Thus, it is clear that R337C variant can produce a dominant negative effect on the transcriptional activity of WT p53 when they are heterologously expressed. Evaluation of transcriptional activity against homotetrameric levels indicated that the R337C homotetramer has very little transcriptional activity.

The R337H variant was identified in patients with Li–Fraumeni‐like syndrome, and this mutation is associated with an increased risk for adrenocortical carcinoma.^[^ ^3^, ^6^ ^]^ The R337H substitution strongly destabilizes the homo‐tetrameric structure,^[^ ^12^ ^]^ but our FRET analysis indicated that the R337H showed about 70% levels of homotetramer formation in comparison to what is measured for the WT p53 homotetramer in cells. Due to the stabilization effect by formation of heterotetramer with WT p53, the **R337H/WT** hetero‐expression showed a slight increase in overall tetramer formation. Consistent with a less stable tetramer relative to WT p53, the transcriptional activity of the R337H variant was reduced to the same level as that of **del/WT** following either homo‐ (**R337H/R337H**) and hetero‐expression (**R337H/WT**) in cells. This indicates that the R337H variant p53 has a dominant‐negative‐like effect, in which its activity is only as high as that of **del/WT**, even when hetero‐expressed in which the expression level of total p53 protein (**R337H/WT**) is double the levels of **del/WT**. The fact that the transcriptional activity was reduced to the same level with both the **R337H/R337H** and **R337H/WT** cases suggests that the **R337H/WT** hetero‐tetramer has the same transcriptional activity as the **R337H/R337H** homo‐tetramer, indicating that the activity is greatly reduced when even a single monomer of R337H p53 is included in the tetramer with three monomers of the WT p53.

The R337P variant of p53 was reported in a single LFS case,^[^ ^13^ ^]^ and it behaved quite differently in our experiments. In previous work, we had shown that when incorporated into a peptide encompassing just the TD of p53, the R337P variant peptides exists predominantly as a monomer.^[^ ^12^ ^]^ Our FRET data in cells confirmed that when the R337P mutant is incorporated in the context of a full‐length protein, it fails to form stable homotetramers which is consistent with our results with the peptides containing the proline substitution at R337 of p53. However, the R337P variant was capable of forming a limited amount of heterotetramer when coexpressed with WT p53. Despite forming some hetero‐tetramers in cells, the R337P consistently exhibited little to no transcriptional activity when expressed homologously, or as hetero‐complexes with WT p53.

The three tetramerization domain variants analyzed in this study, R337C, R337H, and R337P, when heterologously expressed with WT, had their transcriptional activity reduced to either equivalent levels or lower than that of the heterozygous deletion model. All of the variants were capable of forming heterotetramers with WT p53, which suggests that the total amount of tetramer in the cells is greater than **del/WT**, the heterozygous deletion model, when each variant is heterologously expressed with WT p53. This analysis also suggests that the decrease in transcriptional activity is not only due to decreased formation of tetramers, but also to the formation of unstable tetramers that sequester the WT p53 in an inactive or less active form.

Promoter‐specific differences were also evident in our study. The relative transcriptional activity from the *CDKN1A* promoter, normalized by **WT/WT** activity for the *bax* promoter, was increased roughly fourfold. The binding affinities (KD values) for the *CDKN1A* response sequence (4.9 nM at the 5′ site and 12.0 nM at the 3′ site) are substantially lower than that of the *bax* sequence (73 nM).^[^ ^11^ ^]^ These results suggest that promoter‐specific factors, such as intrinsic DNA binding affinity, modulate the impact of R337 variants of p53 on transcriptional activity. More specifically, our results indicate that the R337 variants are not capable of maintaining the stable tetramer required to induce the necessary DNA bending for transcriptional activity, and in particular at promoters regulating apoptosis‐related genes. Furthermore, as shown in Figure 5, the transcriptional activity per unit of FRET signal was substantially lower for **R337C/WT** and **R337H/WT** compared to **WT/WT**. This indicates that even with ≈70% FRET signal, the resulting tetramers are functionally impaired. Thus, partial oligomerization detected by FRET does not guarantee full transcriptional competence. Prior studies have shown that p53 tetramerization induces DNA bending essential for transcriptional activation,^[^ ^14^ ^]^ supporting the idea that destabilized tetramers may fail to induce such structural changes. This mechanistic link is supported by previous structural and biophysical studies showing that p53 binds DNA as a tetramer in a manner that induces specific conformational changes, including minor groove compression and DNA bending. Furthermore, to quantitatively assess this relationship, we plotted normalized transcriptional activity against normalized FRET signal at a fixed expression level (Cerulean intensity 29) for each variant (Figure S3B,C, Supporting Information). Notably, **R337C/WT** and **R337H/WT** showed marked reductions in transcription per unit of oligomerization, reinforcing the idea that structurally compromised tetramers—despite measurable FRET signal—are transcriptionally less efficient. For example, crystallographic analyses have demonstrated that cooperative binding of p53 tetramers induces a characteristic bend in the DNA helix that facilitates transcriptional initiation.^[^ ^14b^ ^]^ Similarly, it has been shown that mutations that impair tetramerization reduce the degree of DNA bending and cooperative DNA engagement.^[^ ^14a^ ^]^ These findings suggest that reduced tetramer stability can indeed hinder the DNA structural remodeling required for robust transcriptional activation. In addition to differences in intrinsic DNA binding affinity, other factors such as promoter architecture, chromatin accessibility, and co‐factor recruitment may also contribute to the observed transcriptional divergence between the *CDKN1A* and *bax* promoters. The *CDKN1A* promoter has been reported to be more readily accessible to p53 binding in several cellular contexts, whereas bax transcription can be more dependent on additional signals or chromatin remodeling.^[^ ^15^ ^]^ These context‐dependent features may modulate the impact of tetramer destabilization on p53‐dependent gene activation at the two different promoters.

Notably, the dominant‐negative‐like effect observed with the R337 variants appears to be less pronounced than what is seen with classical dominant‐negative DBD variants such as the R273H variant of p53. Although the R337 variants reduce transcriptional activity to levels that approach or partially overlap with those seen in the heterozygous deletion condition, the R337H variant in particular retains measurable transcriptional activity, especially at the *CDKN1A* promoter. They do not cause as marked a decrease in overall function as the R273H variant. This distinction may partly explain why germline mutations at R337 are more common than somatic mutations in *TP53*; a milder reduction in p53 activity may permit survival to reproductive age, thereby facilitating inheritance of the mutation.

Finally, loss of heterozygosity (LOH) has been reported in tumor tissues from LFS patients.^[^ ^16^ ^]^ In such cases, when only the mutant allele is expressed, the function of the resulting variant protein is almost completely lost, which likely contributes to carcinogenesis. Indeed, our data show that, aside from R337H variant, which retains minimal activity, even the hetero‐tetramers of the R337C and R337P variants display severely compromised transcriptional activity in cells.

In summary, our study demonstrates that the R337C, R337H, and R337P variants of p53 exert dominant‐negative or dominant‐negative‐like effects that reduce overall transcriptional activity to levels comparable to heterozygous deletion. These findings clarify the role of mutations in the DNA that correspond to the TD domain of p53 and provide novel insights into the molecular mechanisms underlying LFS. Furthermore, we propose that the inability to maintain stable tetramers is necessary for effective DNA bending and transcriptional activation of p53, and is a critical factor in the dominant‐negative‐like effects observed with these R337 variants. Future studies should address the in vivo relevance of these findings and explore strategies to stabilize tetramers formed with variant proteins containing substitution within the TD of p53 as a potential therapeutic approach for LFS. Recent structural and biochemical studies have shown that stabilization of mutant p53 tetramers can rescue transcriptional activity in certain *TP53* variants. For example, small molecules and peptide‐based approaches have been reported to restore tetrameric structure and function in specific p53 mutants.^[^ ^7^, ^17^ ^]^ Our findings suggest that R337 variants, despite retaining the ability to form tetramers, exhibit instability and transcriptional loss, making them potential candidates for such reactivation strategies.

This study has several limitations. The use of overexpression systems and reporter constructs may not fully reflect the regulation of endogenous p53 in physiological settings. Factors such as chromatin context and cofactor availability could influence transcriptional responses differently. Future studies using genome‐edited or endogenous expression models will be necessary to validate these findings.

## 4 Experimental Section

4.1

4.1.1

##### Synthesis of p53TD Peptides

The peptides corresponding to the human WT p53TD (residues 319–358 of human p53); the R337H, R337C, and R337P p53TD variants; and the WT TD (residues 397–444 of human p63) were synthesized as previously described.^[^ ^12^ ^]^ The peptide sequences are shown in Table 1. The synthesized peptides were deprotected and cleaved from the resin by using Reagent K (TFA:H_2_O:thioanisole:ethanedithiol:phenol = 82.5:5:5:2.5:5). All peptides were purified to homogeneity using reverse‐phase HPLC over a C8 column (Vydac, Hsperia, CA, USA) and the correct molecular weight of each peptide was verified by MALDI‐TOF‐MS analysis (Table S1 and Figure S4, Supporting Information). Peptide concentrations were determined using an extinction coefficient, ε_280_ = 1490 M^−1^ cm^−1^, which corresponds to a single Tyr residue present in all of the p53TD and p63TD.

##### Avidin Biotin Pull‐Down Assay for *I* *n* *V* *itro* *Heterotetramer Formation*

For the in vitro avidin‐biotin pull‐down experiments to identify formation of heterotetramers, a biotin‐labeled WT p53TD was mixed with the nonbiotinylated p53TD peptides (WT and variants). To prepare the biotinylated p53TD and p63TD peptides, biotin was conjugated to resin at the N‐terminus of the peptide with two residues of aminocaproic acid (Aca) as a linker by regular solid‐phase peptide synthesis. The Biotin‐(Aca)_2_‐p53TD and Biotin‐(Aca)_2_‐p63 peptides were then dialyzed into a binding buffer consisting of 50 mM Na phosphate buffer (pH 7.5) 100 mM NaCl. For the binding reaction, 40 μL of NeutrAvidin UltraLink Resin (Thermo SCIENTIFIC) beads (50% slurry) was placed in a tube and centrifuged to remove the supernatant. Then, 40 μL of the binding buffer was added and centrifuged to remove the supernatant. This procedure was repeated twice to buffer exchange the beads for the binding reaction. For the binding reaction, the Bio‐(Aca)_2_‐p53TD peptide was added at a concentration of 10–10 μM of the p53TD. The mixed solution was boiled at 100 °C for 5 min and first cooled at room temperature for 1 h and then at 4 °C for an additional 1 h to promote hetero‐oligomerization. Then, 40 μL of buffer‐exchanged beads and 60 μl of buffer were added and incubated at 4 °C for 2 h to bind Bio‐(Aca)_2_‐p53TD to the Avidin beads. The supernatant was removed by centrifugation, washed with 200 μL of binding buffer, and then washed a second time with H_2_O. Next, 200 μL of 80% acetic acid was added and incubated at 4 °C for 2 h to elute the bound peptides from the avidin beads. After centrifugation, 125 μL of the supernatant was mixed with buffer A (0.05% TFA/H_2_O) to make a final solution of 525 μL. If the p53TD sequence contained a Cys, the reducing agent TCEP was added to a final concentration of 1 mM. The solution was then analyzed by reverse‐phase HPLC to determine the amount of each peptide that bound to the resin. A SHISEIDO Proteonavi column (Cat No. 80205) was used for the HPLC analysis of the WT and p63TD experiments and a GL Sciences Inter Sustain C8 column (Cat No.5020‐16021) was used for the HPLC analysis of the R337C, R337H, and R337P experiments. The results were expressed as a ratio of either the variant p53TD or p63TD pulled down with respect to the amount of biotinylated p53TD.

##### Cells Used in the Experiments

p53‐null lung cancer cell line H1299 (RRID: CVCL_0060; obtained from American Type Culture Collection) derived from human lung cancer was used for the analysis. Cells were cultured in RPMI‐1640 (SIGMA) medium with 10% Fetal Bovine Serum (FBS; Thermo Scientific) and penicillin–streptomycin (Gibco) (penicillin (100 units mL^−1^), streptomycin (100 μg mL^−1^)). Culture conditions were 37 °C 5% CO_2_.

##### Plasmid Construction

Plasmids for expressing p53 protein fused with fluorescent proteins for FRET assay used in this study were as follows

phCMV‐p53(WT)‐Venus‐2A‐p53(WT)‐Cerulean (**WT/WT**);

phCMV‐p53(3Ala)‐Venus‐2A‐p53(3Ala)‐Cerulean (**3Ala/3Ala**);

phCMV‐p53(3Ala)‐2A‐p53(WT)‐Cerulean (**3Ala/WT**);

phCMV‐Venus‐NLS‐2A‐p53(WT)‐Cerulean (**del/WT**);

phCMV‐p53(R273H)‐Venus‐2A‐p53(R273H)‐Cerulean (**R273H/R273H**);

phCMV‐p53(R273H)‐Venus‐2A‐p53(WT)‐Cerulean (**R273H/WT**);

phCMV‐p53(R337H)‐Venus‐2A‐p53(R337H)‐Cerulean (**R337H/R337H**);

phCMV‐p53(R337H)‐Venus‐2A‐p53(WT)‐Cerulean (**R337H/WT**);

phCMV‐p53(R337C)‐Venus‐2A‐p53(R337C)‐Cerulean (**R337C/R337C**);

phCMV‐p53(R337C)‐Venus‐2A‐p53(WT)‐Cerulean (**R337C/WT**);

phCMV‐p53(R337P)‐Venus‐2A‐p53(R337P)‐Cerulean (**R337P/R337P**);

phCMV‐p53(R337P)‐Venus‐2A‐p53(WT)‐Cerulean (**R337P/WT**).

If p53‐Venus and p53‐Cerulean form hetero‐tetramer, FRET signal between Venus and Cerulean can be observed in this system.

For the p53 reporter plasmids, pp53RE(*bax*)‐mCherry‐NLS‐AU2 and pp53RE(*CDKN1A*)‐mCherry‐NLS‐AU2 were used in this study. There are two different types of p53‐responsive elements used for the reporter site. The p53RE(*bax*), a responsive element present in the apoptosis‐inducing *bax* gene (Figure S5A, Supporting Information), and the p53RE(*CDKN1A*), a responsive element from the *CDKN1A* gene (Figure S5B, Supporting Information). In Figure S5A and S5B, Supporting Information, the underlined bases indicate the consensus p53‐binding sites, whereas the capital letters indicate palindromic consensus sequences that bind to p53. Plasmid constructs generated in this study, including the dual‐tagged FRET vectors, are available from the corresponding author upon reasonable request.

##### Measurement of Intracellular p53 Tetramerization and Transcriptional Activity

H1299 cells were seeded at a 3.0 × 10^5^ cells dish^−1^ density in a 35 mm diameter tissue culture dish. Twenty‐four hours later, 1.2 μg of each type of plasmid DNA (p53 expression plasmid and reporter plasmid) was added to 100 μL of Opti‐MEM, mixed with 6 μL of lipofectamine 2000 (Thermo Fisher Scientific), and left at room temperature for 10 min. After washing twice with 1.5 mL of PBS per dish, the mixture was combined with 700 μL of RPMI‐1640 (without 10% FBS P/S), added to the dish, and incubated at 37 °C, 5% CO_2_ for 1 h. After the incubation, the mixture of RPMI‐1640 (without 10% FBS P/S) and lipofectamine 2000 was removed, and 1.5 mL of RPMI‐1640 (without 10% FBS P/S) was added, followed by incubation at 37 °C and 5% CO_2_. After 11 h, the medium was removed, 700 μL of 10% formalin PBS solution was added, and the cells were shaken in an invitroshaker (TAITEC) for 10 min. Then, the 10% formalin solution was removed, and 700 μL of 0.2% Triton‐X‐100 PBS solution was added, followed by shaking at room temperature for 10 min. Next, 0.2% Triton‐X‐100 PBS solution was removed and washed three times with PBS, and 1 mL of 1000‐fold diluted DAPI PBS solution was added to the dish and shaken for 10 min. Then, another 1 mL of PBS was added.

A BIOREVO BZ‐9000 fluorescence microscope (Keyence) was used for observation. The fluorescence mirror units used were YFP‐BP for Venus, CFP‐2342C for Cerulean, DAPI‐BP for DAPI, TRITC for mCherry, and FRET for FRET observation. A Nikon Plan Fluor D 10×/NA0.30 ph 1 objective lens was used, and the exposure times were FRET 1/2 s, Cerulean 1/2 s, DAPI 1/4 s, Venus 1/2 s, and mCherry 1/2 s. The images were acquired at 8 bits and 5 × 6 fields of view, shifted by one field of view from the center, and were acquired in the order of upper right, lower right, lower left, and upper left, centered on the (0, 0) point. After the DAPI, Venus, FRET, and Cerulean fluorescence images were acquired in this order, the FRET mirror unit was changed to TRITC, and only mCherry fluorescence images were acquired in 4 planes of 30 consecutive fields of view.

##### Image Analysis Methods

Data quantitative analysis of fluorescence intensity was performed as previously described.^[^ ^18^ ^]^ For Venus, Cerulean, and mCherry, the average of the raw data without subtracting the background value for each pixel in the cell nucleus detected by DAPI is taken; for FRET, the FRET fluorescence intensity for each pixel in the nucleus is calculated from the FRET fluorescence intensity of the raw data. The FRET signal value was calculated by subtracting Venus × 0.16 and Cerulean × 0.33, which are contributions from the fluorescence of Venus and Cerulean alone, from the FRET fluorescence intensity of the raw data per pixel per nucleus, and the average of these values per nucleus was taken as the FRET signal value. This is used to calculate the average FRET signal to Cerulean Intensity, the average mCherry Intensity, and the mCherry Intensity to FRET signal for a group of cells with a Venus: Cerulean fluorescence intensity ratio of 1:1. FRET signal to Cerulean Intensity and mCherry Intensity to FRET signal were calculated.

##### Protein Purification

cDNAs corresponding to human wild‐type and R337 variant p53 (R337H and R337C) incorporating the thermostable and cysteine‐modified mutations (C124A, C135V, C141V, W146Y, C182S, V203A, R209P, C229Y, H233Y, Y234F, N235K, Y236F, T253V, N268D, C275A, C277A, K292C) were synthesized by GeneArt (Thermo Fisher Scientific) with flanking BamHI and SalI restriction enzyme sites and cloned into a modified pGEX‐2T vector (Amersham) with a Tobacco Etch Virus (TEV) protease cleavage site replacing the original thrombin cut site. These proteins were expressed in BL21(DE3)pLysS *E. coli* cells and purified via affinity chromatography. Cells were cultured in 1 L of 2× YT medium at 37 °C. When the culture reached an OD_6__0__0_ of 0.4–0.6, protein expression was induced by the addition of 100 μM IPTG and 100 μM ZnCl_2_. The cells were then cultured at 20 °C for 20 h and harvested by centrifugation. The pellets were resuspended in a Lysis buffer containing 10 mM Na_2_HPO_4_, 1.8 mM KH_2_PO_4_, 2.7 mM KCl, 650 mM NaCl, 1 mM DTT, and 10 mM MgCl_2_ and lysed using a French press. The lysate was centrifuged, and the p53 proteins were purified using glutathione resin (GE Healthcare). The supernatant was removed by centrifugation 3000× g for 5 min and washed twice with Lysis buffer and twice with TEV buffer (20 mM sodium phosphate, pH7.4, 125 mM NaC1, 5 mM DTT). p53 was cleaved from the GST tag by TEV protease on‐column and further purified using a heparin column (HiTrap Heparin HP, GE Healthcare) (with buffer A: 20 mM sodium phosphate, pH7.4; buffer B: 20 mM sodium phosphate, pH7.4, 2 M NaCl) (Figure S6, Supporting Information).

##### Gel Shift Assay

The gel shift assay was performed with Cy3‐labeled *bax* and *CDKN1A* promoter DNA. A single‐stranded oligonucleotide containing the *bax* binding site (5′‐TGGCTCACAAGTTAGAGACAAGCCTGGGC‐3′) and the *CDKN1A* binding site (5′‐GTCAGGAACATGTCCCAACATGTTGAGCTC‐3′) were labeled with Cy3 at the 5′‐end. p53 protein was incubated with 10 nM of annealed double‐strand DNA probe for 1 h at 4 °C in 10 mM Tris‐HCl at pH 7.4 containing 30% (v/v) glycerol or 0.5× Tris‐borate‐EDTA buffer (50 mM Tris‐HCl, 50 mM borate, 1 mM EDTA, pH 8.3) containing 30% (v/v) glycerol. Free and p53‐bound DNA probes were separated by electrophoresis. Reaction mixtures were loaded onto a prerun 4% nondenaturing polyacrylamide gel (the ratio of acrylamide/bisacrylamide = 1:19) containing 0.5× Tris‐borate‐EDTA buffer (50 mM Tris‐HCl, 50 mM borate, 1 mM EDTA, pH 8.3) and 3% (v/v) glycerol. Electrophoresis was carried out at 125 V for 120 min at 4 °C. Signals were detected using an FLA‐3000 luminoimage analyzer (Fujifilm, Tokyo, Japan).

##### Statistical Analysis

Statistical analysis was conducted employing the Kruskal–Wallis test with Dunn's multiple comparison post hoc tests for transcriptional activity and FRET analysis or Student's *t*‐test for pull‐down assays utilizing GraphPad Prism (Version 9.5.1, GraphPad Software, Inc, San Diego CA). A *p*‐value of <0.05 was considered indicative of statistical significance for all comparisons. Significance levels were denoted as *p < 0.05; **p < 0.01; ***p < 0.001; ****p < 0.0001; n.s., not significant.

## Conflict of Interest

The authors declare no conflict of interest.

## Supporting information

Supplementary Material

## Data Availability

Materials including plasmids and cell lines used in this study, are available upon reasonable request.
