# Supplementary material for: Dominant‐Negative Effects of p53 R337 Variants in Li–Fraumeni Syndrome: Impact on Tetramer Formation and Transcriptional Activity
Source: Chembiochem. 2025 Jul 24;26(22):e202500330. doi: 10.1002/cbic.202500330 (PMC12631009; doi:10.1002/cbic.202500330)
Supplement: Supplementary file 1 — Supplementary Material [file CBIC-26-e202500330-s001.pdf]

Supplementary Information

Dominant-Negative Effects of p53 R337 Variants in Li–Fraumeni Syndrome: Impact on Tetramer Formation and Transcriptional Activity

Rui Kamada+[a], Shuya Sakaguchi+ [a], Madoka Kanno[a], Takaaki Ozawa[a], Natsumi Nakagawa[a], James G. Omichinski[b], and Kazuyasu Sakaguchi*[a]

[a] Dr. R. Kamada,+ Dr. S. Sakaguchi,+ Ms. M. Kanno, Mr. T. Ozawa, Dr. N. Nakagawa, Prof. K. Sakaguchi

Laboratory of Biological Chemistry, Department of Chemistry, Faculty of Science, Hokkaido University

Sapporo 060-0810, Japan

E-mail: kazuyasu@sci.hokudai.ac.jp

[b] Prof. J. G. Omichinski

Département de Biochimie et Médicine Moléculaire, Université de Montréal, C.P. 6128 Succursale Centre-Ville, Montréal, QC H3C 3J7, Canada

[+] These authors equally contributed to this work.

Supporting information for this article is given via a link at the end of the document.

Contents:

Figure S1 Representative fluorescence microscopy images of cells expressing dual-tagged p53 constructs.
Figure S2. Analysis of p53 mutant proteins using the p53RE(*CDKN1A*)
Figure S3. Relative transcriptional activity and oligomer formation for each p53 variant.
Table S1. Identification of synthesized peptides by MALDI-TOF/MS.
Figure S4. HPLC chart (Left) and MALDI-TOF MS (Right) spectra of the synthesized peptides. (A,B) p53TD-WT, (C,D) p53TD-R337C, (E,F) p53TD-R337H, (G,H) p53TD-R337P, (I,J) Bio-p53TD, (K,L) p63TD
Figure S5. *bax and CDKN1A* response element sequences
Figure S6. The SDS-PAGE analysis of purified wild-type and R337 mutant-p53 proteins.

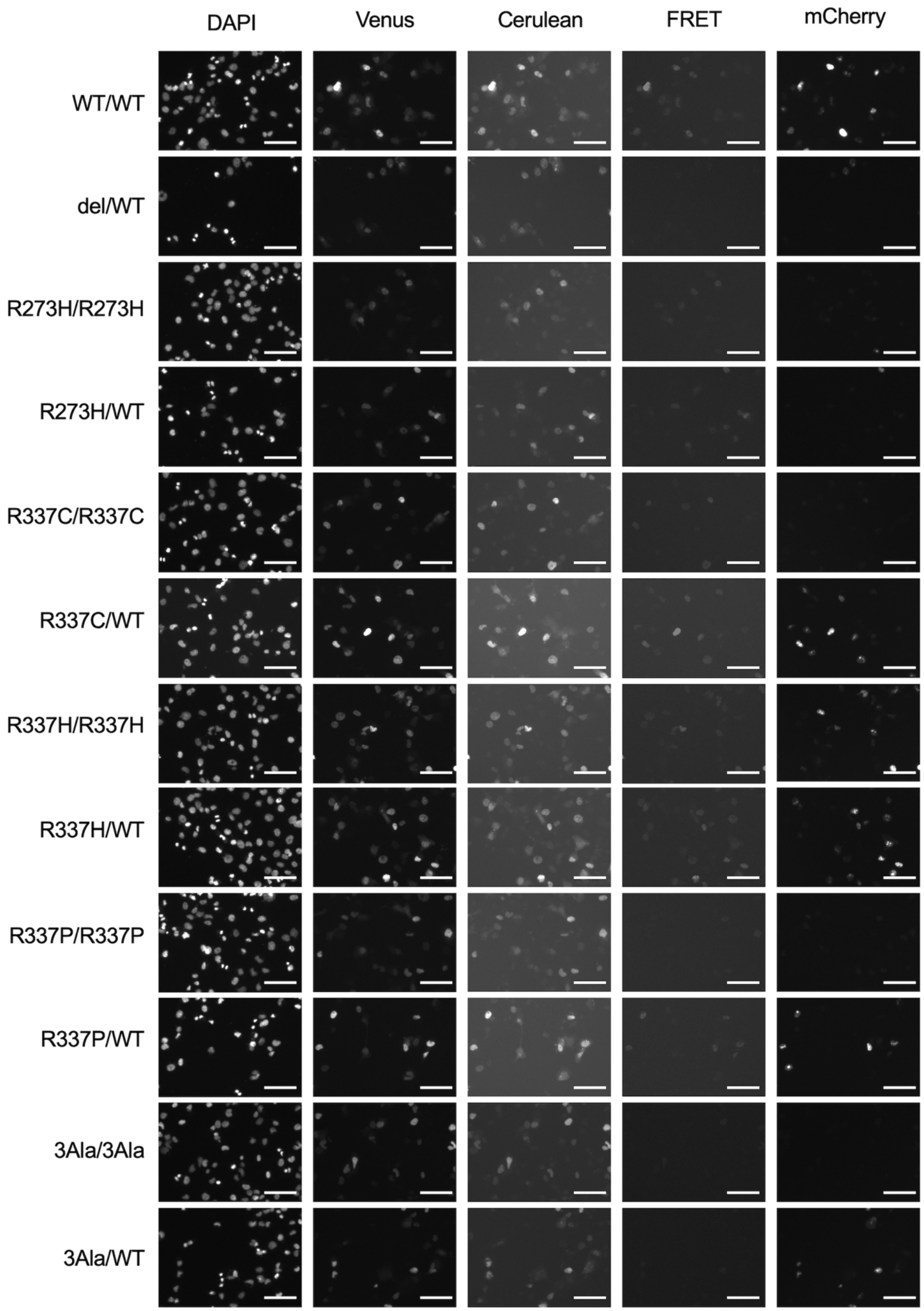

Figure S1. Representative fluorescence microscopy images of cells expressing dual-tagged p53 constructs. Each row shows five fluorescence channels: DAPI (nuclei), Cerulean (donor), Venus (acceptor), FRET signal (oligomer formation), and mCherry (transcriptional activity). The

FRET signal was corrected by subtracting the bleed-through from the donor and acceptor channels (Cerulean × 0.33 and Venus × 0.16, respectively). Representative images are shown for the following combinations of p53 variants: WT/WT, del/WT, R273H/R273H, R273H/WT, R337C/R337C, R337C/WT, R337H/R337H, R337H/WT, R337P/R337P, R337P/WT, 3Ala/3Ala, and 3Ala/WT. These images illustrate the expression patterns and subcellular localization of the dual-tagged constructs, and visually support the quantification of oligomer formation and transcriptional activity presented in the main figures. Scale bar: 100 µm.

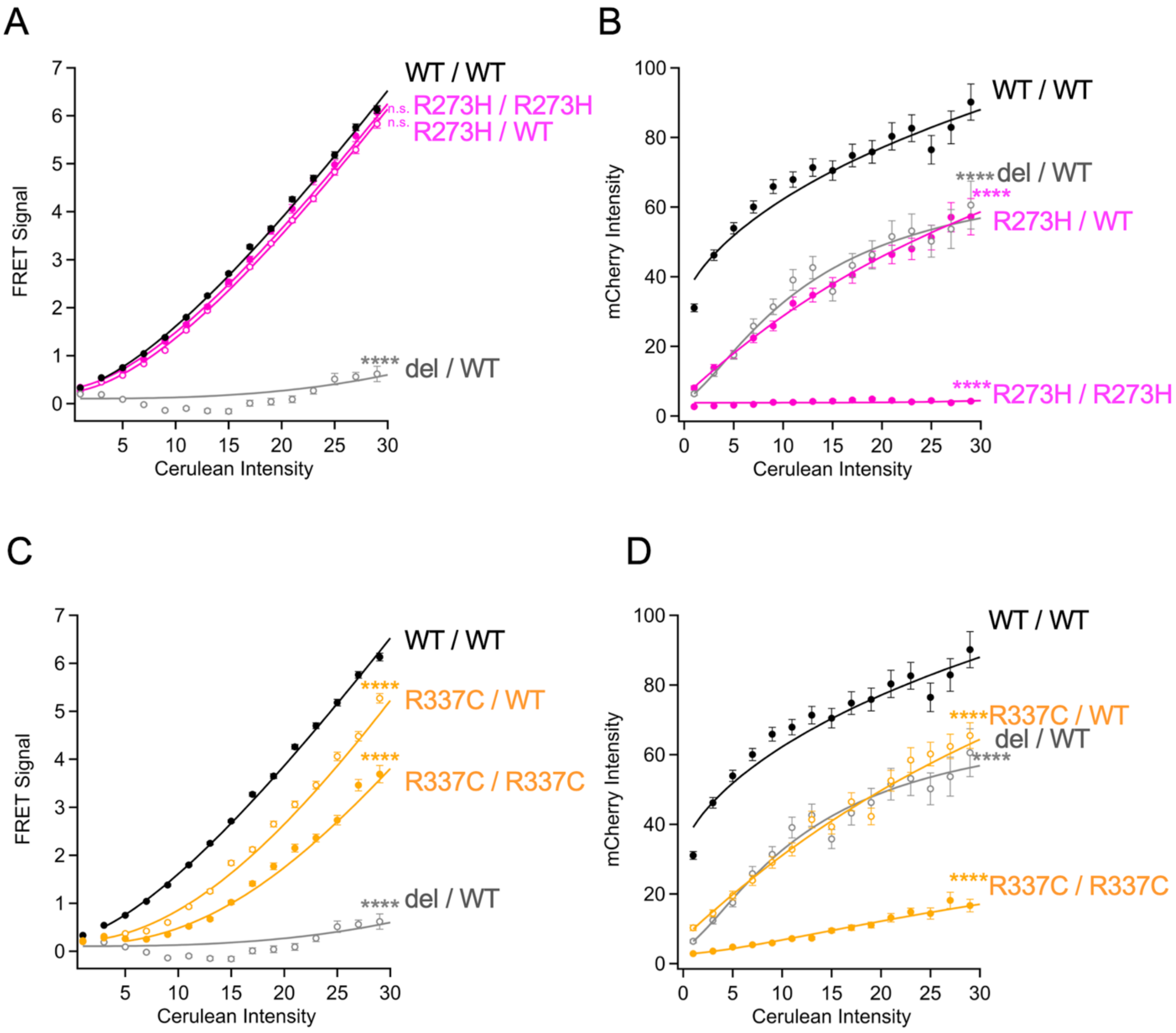

Figure S2 . Analysis of p53 mutant proteins using the p53RE(*CDKN1A*)

Cells were transfected with phCMV-p53(mutant)-Venus-2A-p53(WT)-Cerulean (**Mut/WT**) and phCMV-p53(mutant)-Venus-2A-p53(mutant)-Cerulean (**Mut/Mut**) and analyzed for formation of p53 either homo- or hetero-tetramers (A, C, E, G) as well as transcriptional activity for the pp53RE(*CDKN1A*)-mCherry-NLS-AU2 (B, D, F, H). (A,B) R273H, (C,D) R337C, (E,F) R337H, (G,H) R337P. **WT/WT** and **del/WT** p53 protein were shown in black and grey, respectively. The FRET signals (oligomer formation) and the mCherry (p53-dependent transcription) fluorescence signals in each single cell were quantified. Data represent the mean ± SEM from more than 1,000 cells per condition, based on three independent experiments. Significance was analyzed using the Kruskal-Wallis test. ****p < 0.0001; n.s., not significant.

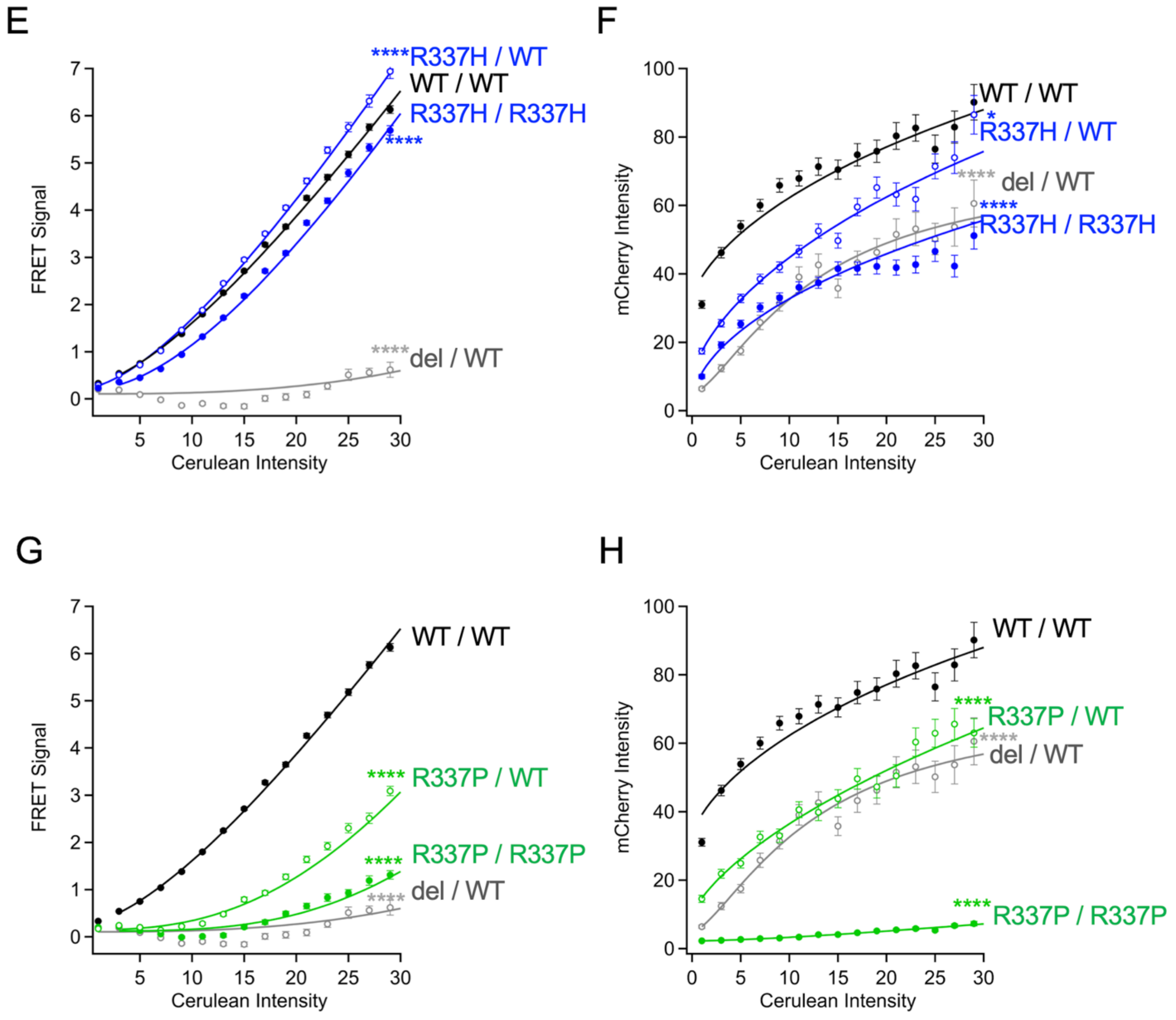

Figure S2 . Analysis of p53 mutant proteins using the p53RE(*CDKN1A*)

Cells were transfected with phCMV-p53(mutant)-Venus-2A-p53(WT)-Cerulean (**Mut/WT**) and phCMV-p53(mutant)-Venus-2A-p53(mutant)-Cerulean (**Mut/Mut**) and analyzed for formation of p53 either homo- or hetero-tetramers (A, C, E, G) as well as transcriptional activity for the pp53RE(*CDKN1A*)-mCherry-NLS-AU2 (B, D, F, H). (A,B) R273H, (C,D) R337C, (E,F) R337H, (G,H) R337P. **WT/WT** and **del/WT** p53 protein were shown in black and grey, respectively. The FRET signals (oligomer formation) and the mCherry (p53-dependent transcription) fluorescence signals in each single cell were quantified. Data represent the mean ± SEM from more than 1,000 cells per condition, based on three independent experiments. Significance was analyzed using the Kruskal-Wallis test. *p < 0.05; ****p < 0.0001.

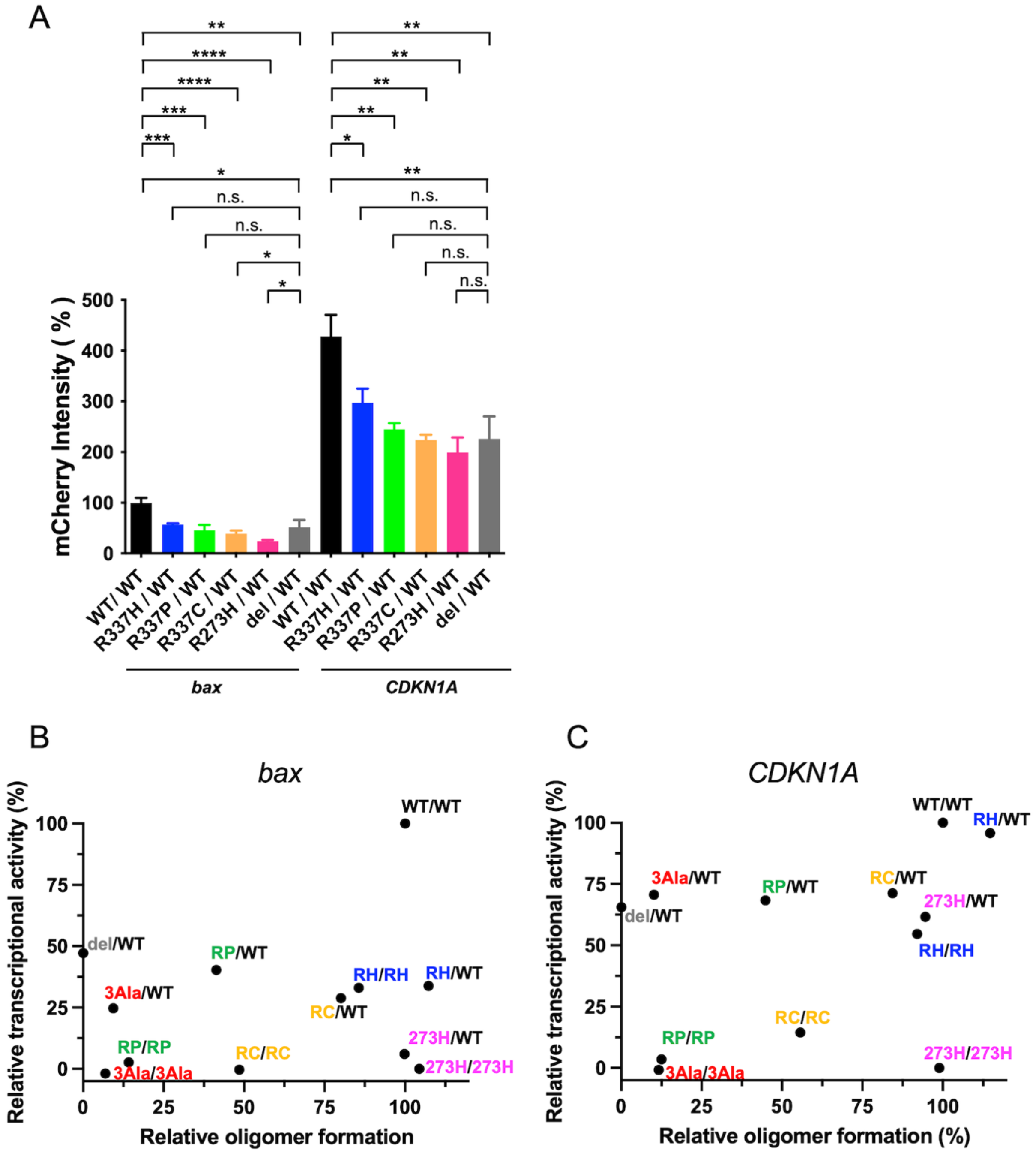

Figure S3. Relative transcriptional activity and oligomer formation for each p53 variant. (A) Transcriptional activity values (mCherry signal) for the *bax* promoter are from Figure 3, 4, and values for the *CDKN1A* promoter are from Supplementary Figure S2 at Cerulean intensity levels of 10-20. All data were normalized to WT/WT activity for the *bax* promoter. The **WT/WT** homo-tetramer with the *CDKN1A* promoter was ~410% more active than that with the *bax* promoter, and **del/WT** was 4.1 times more active.

The hetero deletion model **del/WT** was 50% active with the *bax* promoter and 230% active with *CDKN1A*, while **R337H/WT** was 50% active with the *bax* promoter, comparable to **del/WT**, and

slightly active with the *CDKN1A* promoter, showing 300% activity. **R337P/WT** and **R337C/WT** had similar transcriptional activity to **del/WT**, at 45% and 250%, and 40% and 240%, respectively. Data represent the mean ± SD from at least three independent experiments. Significance was analyzed using Student's t-test. * $p < 0.05$; **$p < 0.01$; n.s., not significant. (B, C) Correlation between normalized p53 oligomer formation and transcriptional activity for each variant, using data from the *bax* promoter (B) and the *CDKN1A* promoter (C). FRET signal (x-axis) and mCherry signal (y-axis) were obtained from the Cerulean intensity point 29. FRET values were normalized to 100% for WT/WT and 0% for del/WT. mCherry values were normalized to 100% for WT/WT and 0% for R273H/R273H. Each point represents a distinct p53 variant, illustrating the relative transcriptional activity per unit of oligomer formation. These data indicate that partial retention of oligomerization does not necessarily result in a proportional amount of transcriptional activity.

Table S1. Identification of synthesized peptides by MALDI-TOF/MS.

Peptides	Calculated ($M+H^+$)	Observed
WT	4785.51	4785.71
R337C	4732.47	4733.45
R337H	4766.47	4766.64
R337P	4726.44	4726.42
Bio-p53Tet	5238.16	5238.18
p63Tet	5962.82	5963.78

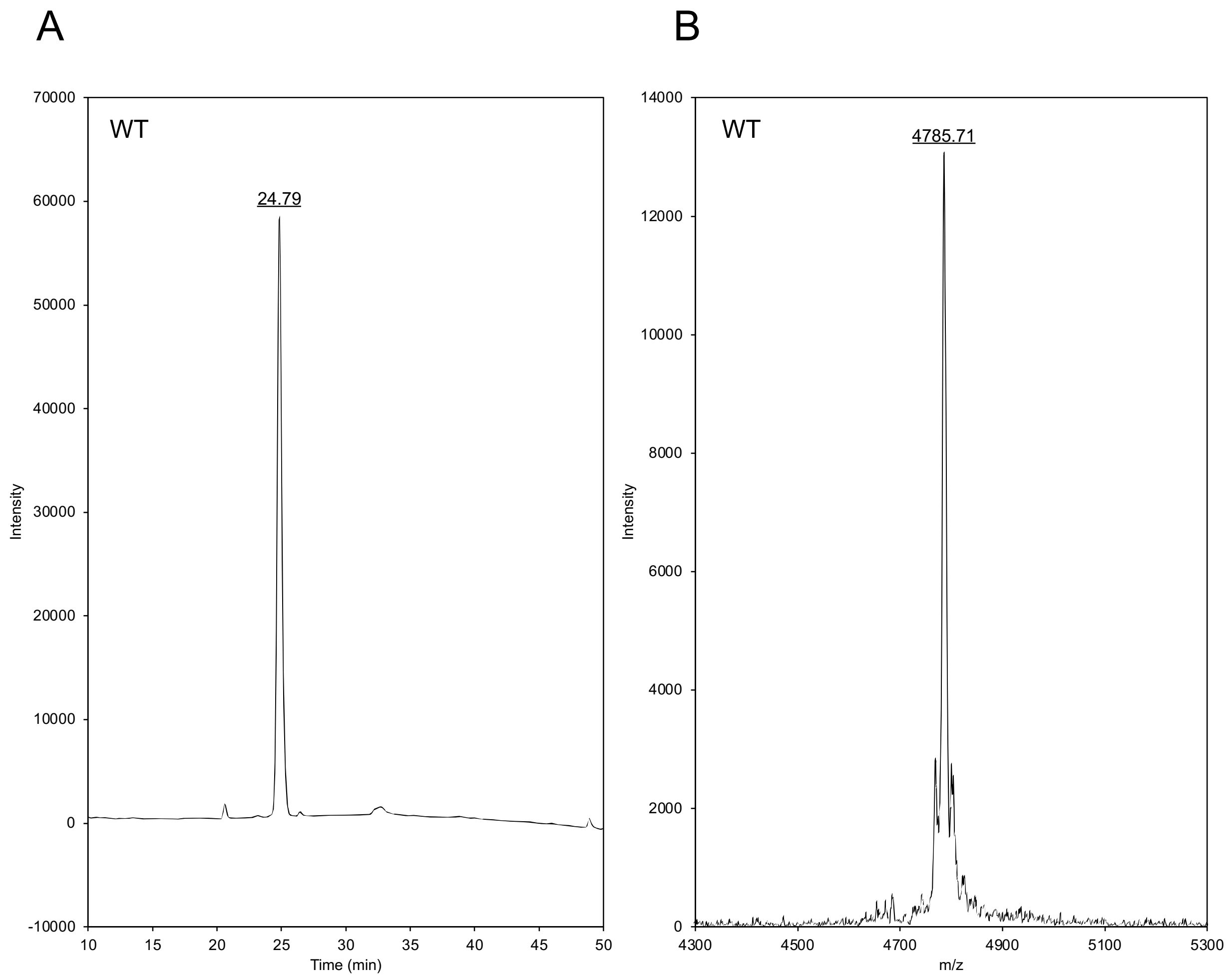

Figure S4. HPLC chart (Left) and MALDI-TOF MS (Right) spectra of the synthesized peptides. (A,B) p53TD-WT, (C,D) p53TD-R337C, (E,F) p53TD-R337H, (G,H) p53TD-R337P, (I,J) Bio-p53TD, (K,L) p63TD

C

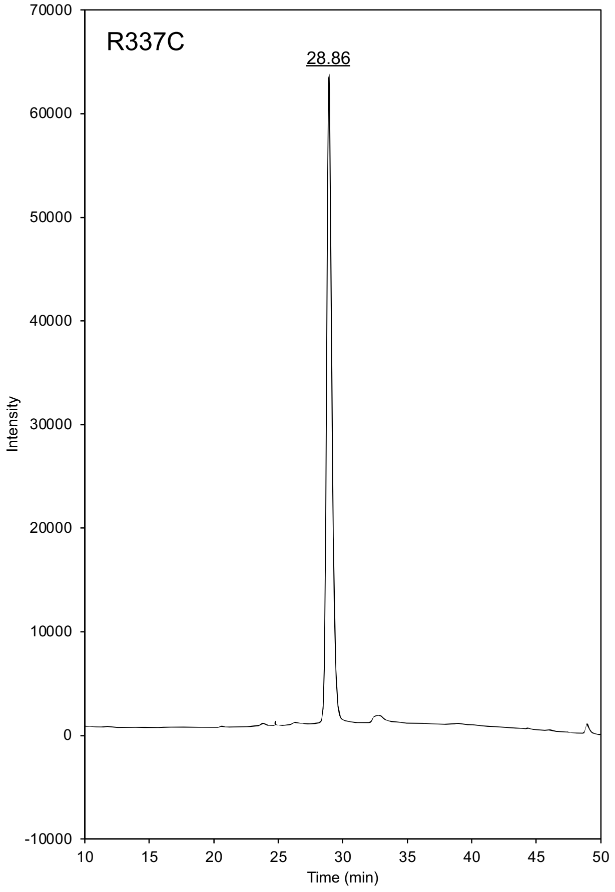

D

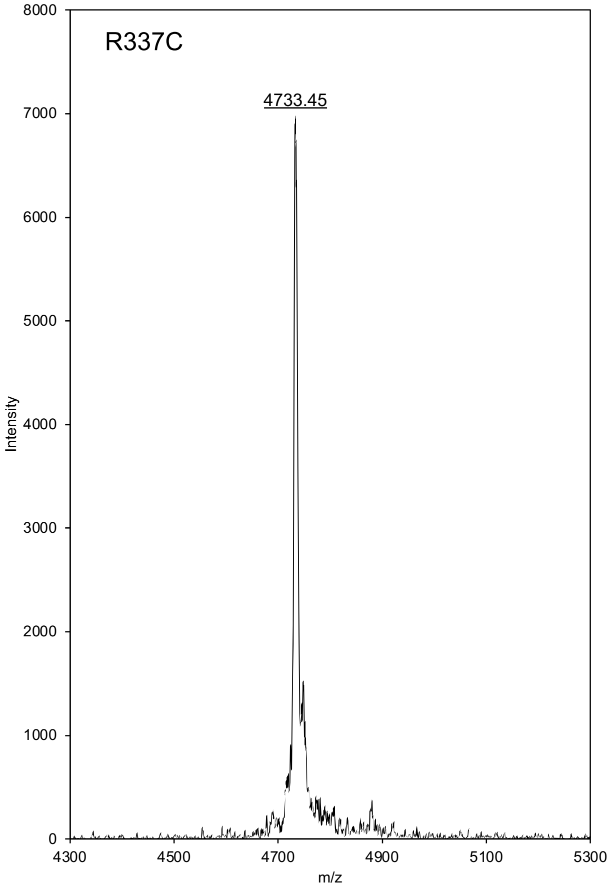

Figure S4. HPLC chart (Left) and MALDI-TOF MS (Right) spectra of the synthesized peptides. (A,B) p53TD-WT, (C,D) p53TD-R337C, (E,F) p53TD-R337H, (G,H) p53TD-R337P, (I,J) Bio-p53TD, (K,L) p63TD

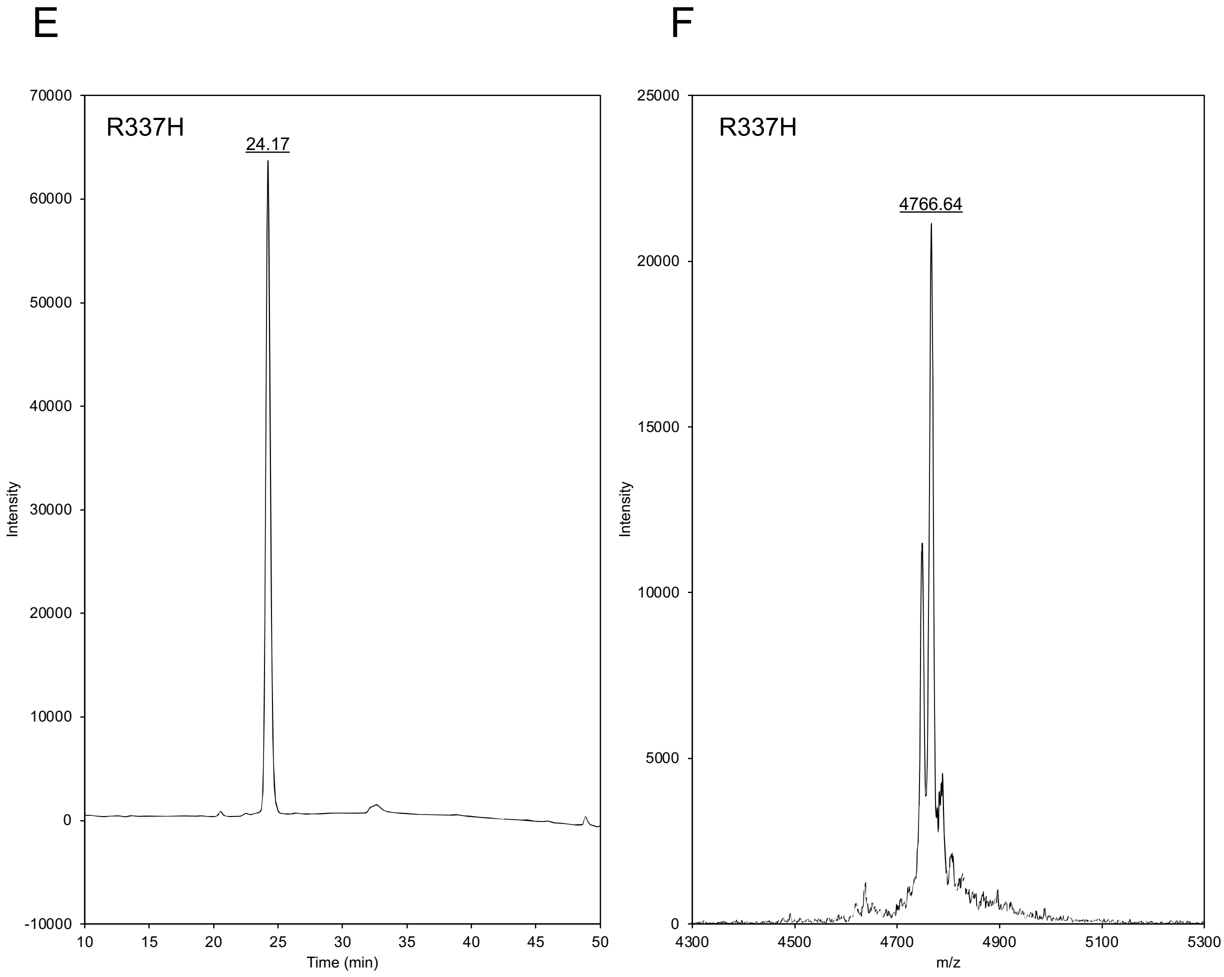

Figure S4. HPLC chart (Left) and MALDI-TOF MS (Right) spectra of the synthesized peptides. (A,B) p53TD-WT, (C,D) p53TD-R337C, (E,F) p53TD-R337H, (G,H) p53TD-R337P, (I,J) Bio-p53TD, (K,L) p63TD

G H

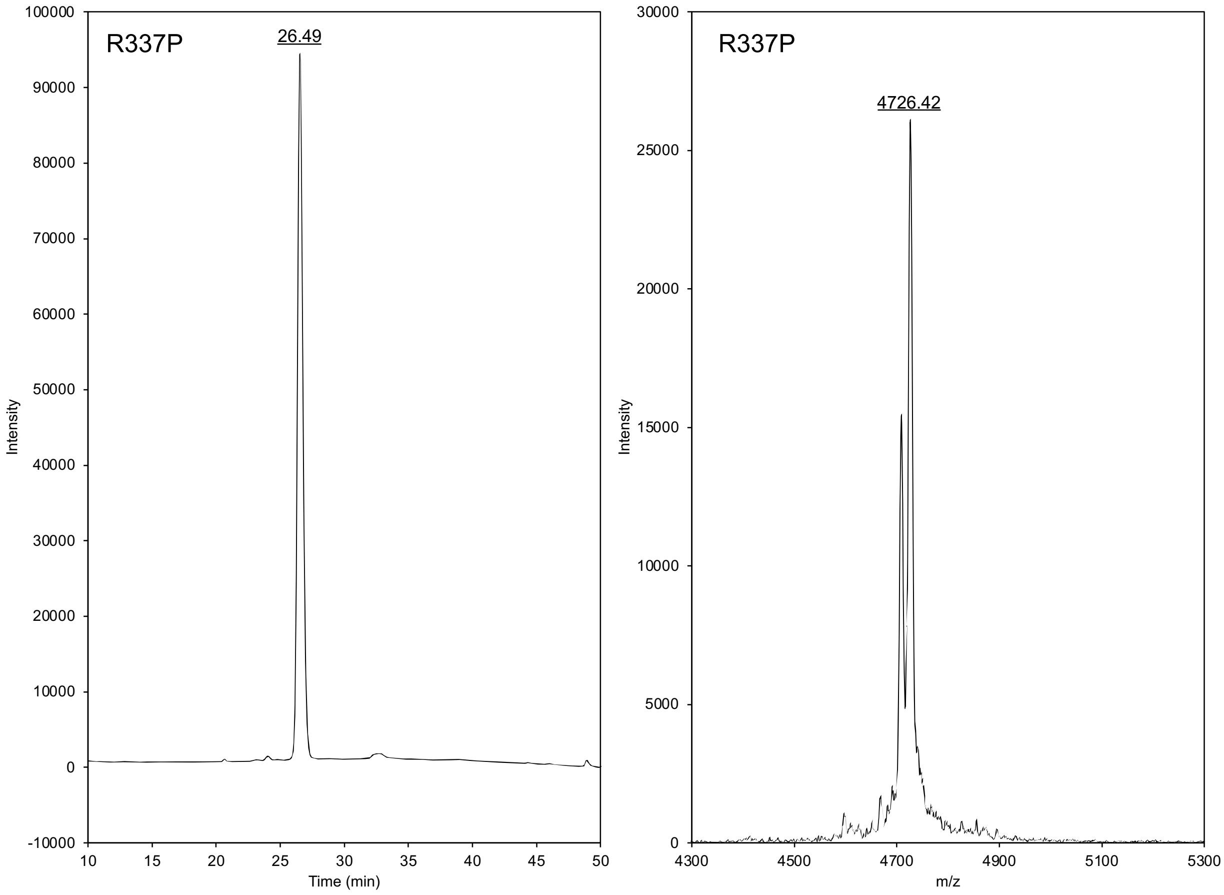

Figure S4. HPLC chart (Left) and MALDI-TOF MS (Right) spectra of the synthesized peptides. (A,B) p53TD-WT, (C,D) p53TD-R337C, (E,F) p53TD-R337H, (G,H) p53TD-R337P, (I,J) Bio-p53TD, (K,L) p63TD

I

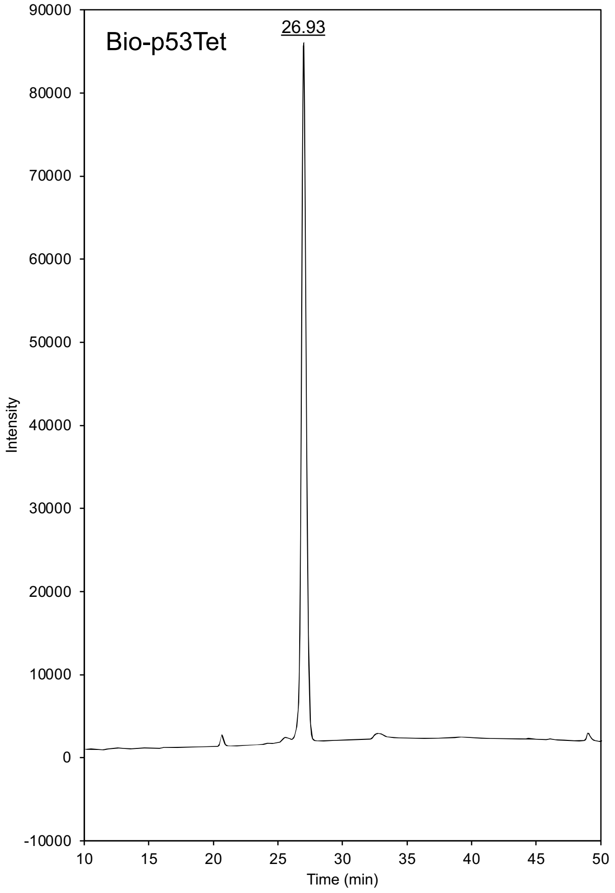

J

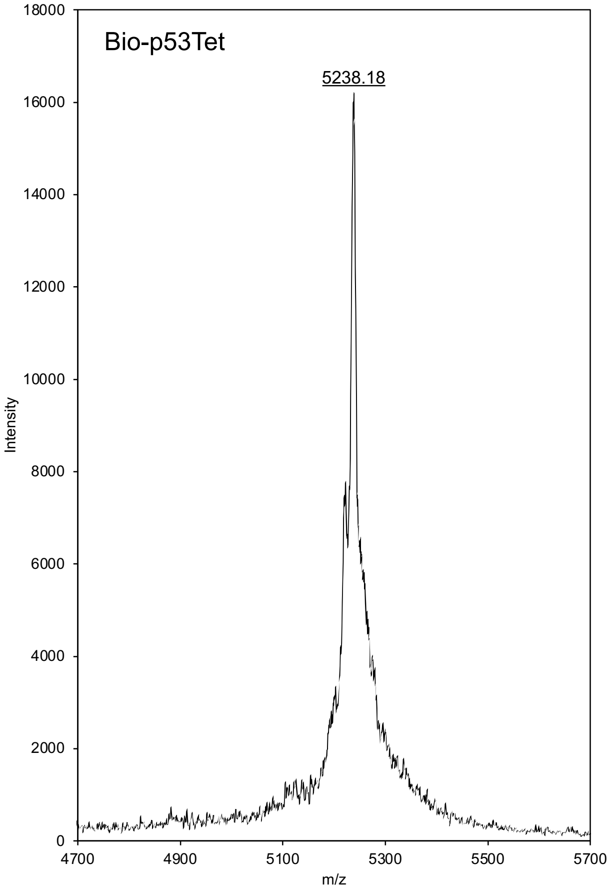

Figure S4. HPLC chart (Left) and MALDI-TOF MS (Right) spectra of the synthesized peptides. (A,B) p53TD-WT, (C,D) p53TD-R337C, (E,F) p53TD-R337H, (G,H) p53TD-R337P, (I,J) Bio-p53TD, (K,L) p63TD

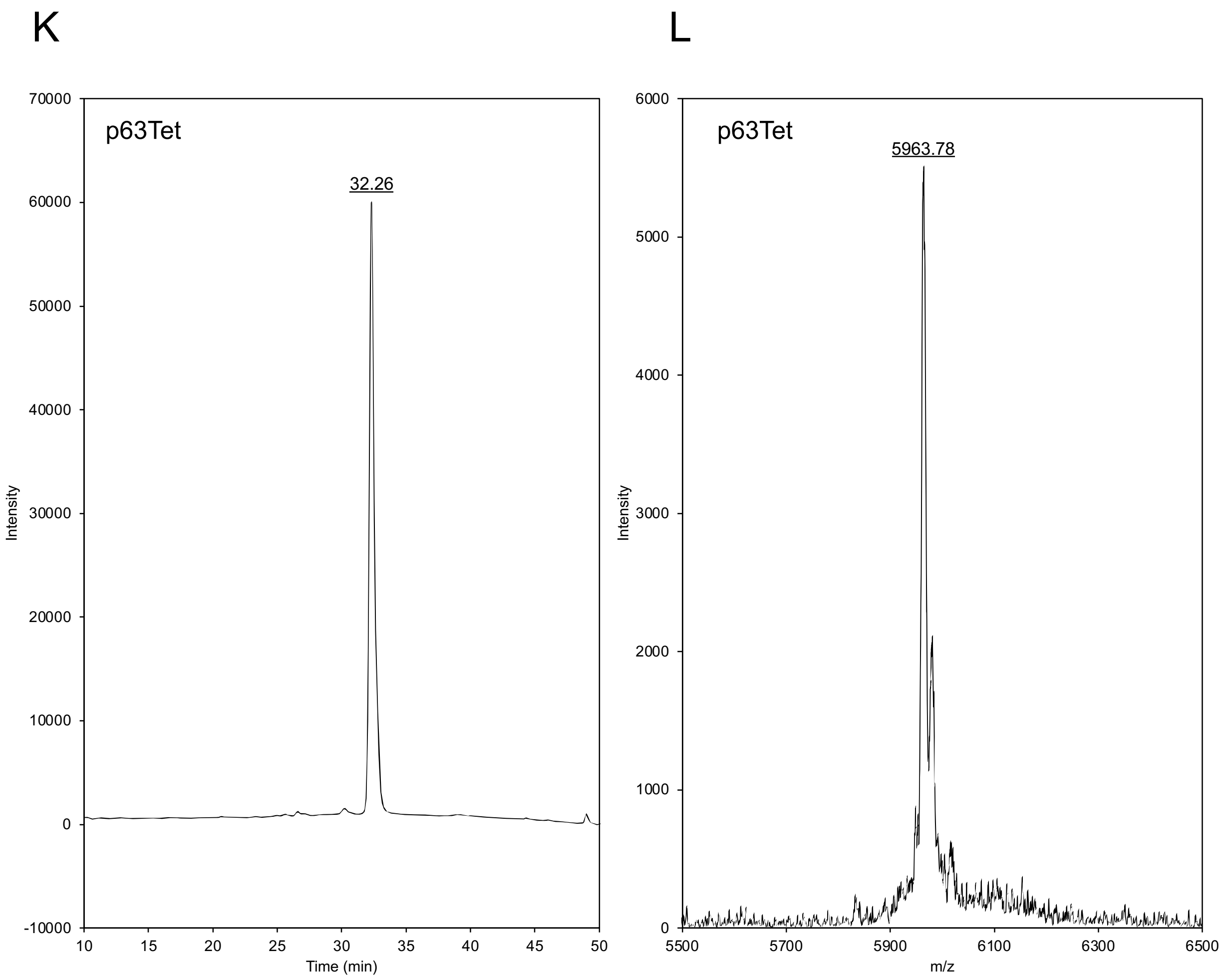

Figure S4. HPLC chart (Left) and MALDI-TOF MS (Right) spectra of the synthesized peptides. (A,B) p53TD-WT, (C,D) p53TD-R337C, (E,F) p53TD-R337H, (G,H) p53TD-R337P, (I,J) Bio-p53TD, (K,L) p63TD

A

bax (565 bp)

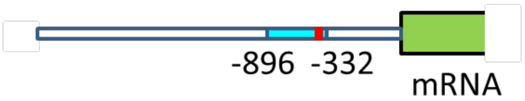

(−896)

```
ccctgctgat ctatcagcac agattagttt ctgccacttt ttaaacttca
tattcctttt ctttttacac aaacacaaac attcgagtca tgactgggtg
gggtggctca agcctgtaat ctcagcactt tgggaggcca aggtgcgagg
atcgcttgag tctgggagtt cagagaccag cctgggcaac atagagagac
ctcatctcca cataaaaagt tttaaaaatt aaccaggggc ggtgtagtcc
cagctactca ggaggctgag gtgggaggct tcagcccggg aattccagac
tgcagtgagc catgattggg ccactgcact ccagcctggg caacacagtg
agaccctgtc tcaaaaaaaa aaaaaaaaaa aacaggaaaa aacaaacaaa
cagaaaagca ggcctggcgc ggtagctcat gcctgtaatc ccagcgcttt
ggaaggctga gacggggtta tctcttgggc tcACAAGTTa gAGACAAGCC
TGGGCgTggg CTAtaTTgct agatccaggt ctctgcaaaa aacaaaacca
ctcagttttt agtca
```

(−332)

Figure S5. ***bax and CDKN1A*** **response element sequences**
The DNA sequence of the response element from the *bax* (A) and *CDKN1A* (B) gene is shown. Red letters indicate the p53-binding region, and the underlined capital letters indicate the palindromic consensus p53-binding sequence. The number of bases from the transcription start point are indicated in parentheses.

B

CDKN1A(1014 bp)

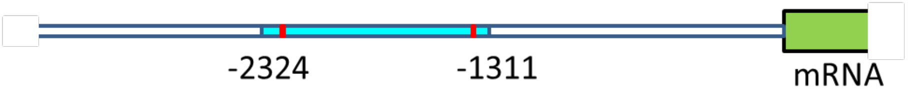


```
(−2324)
cctgcttccc aggaacatgc ttgggcagca ggctgtggct ctgattggct
ttctggccgt caGGAACATG TCCcAACATG TTGagctctg gcatagaaga
ggctggtggc tattttgtcc ttgggctgcc tgttttcagg tgaggaaggg
gatggtagga gacaggagac ctctaaagac cccaggtaaa ccttagcctg
ttactctgaa cagggtatgt gatctgccag cagatccttg cgacagggct
gggatctgat gcatgtgtgc ttgtgtgagt gtgtgctggg agtcagattc
tgtgtgtgac ttttaacagc ctgctccctt gcctttttca gggcagaagt
cctcccttag agtgtgtctg ggtacacatt caagtgcatg gttgcaaact
ttttttttta aagcactgaa tagtactaga cacttagtag gtacttaaga
aatattgaat gtcgtggtgg tggtgagcta gaagttataa aaaaaattct
ttcccaaaaa caacaacaaa aagaattatt tcattgtgaa gctcagtacc
acaaaaattt aaataattca ttacaagcct ttattaaaaa aaatttctc
cccaaagtaa acagacagac aatgtctagt ctatttgaaa tgcctgaaag
cagaggggct tcaaggcagt gggagaaggt gcctgtcctc tgctggacat
ttgacaacca gccctttgga tggtttggat gtataggagc gaaggtgcag
acagcagtgg ggcttagagt ggggtcctga ggctgtgccg tggcctttct
ggggtttagc cacaatcctg gcctgactcc agggcgaggc aggccaaggg
ggtctgctac tgtgtcctcc cacccctacc tgggctccca tccccacagc
agaggagaaa gaagcctgtc ctccccgagg tcagctgcgt tagagGAAgA
AGaCTGGGCA TGTCTgggca gagatttcca gactctgagc agcctgagat
gtcagtaatt gtag
(−1311)
```

Figure S5. ***bax and CDKN1A*** **response element sequences**
The DNA sequence of the response element from the *bax* (A) and *CDKN1A* (B) gene is shown. Red letters indicate the p53-binding region, and the underlined capital letters indicate the palindromic consensus p53-binding sequence. The number of bases from the transcription start point are indicated in parentheses.

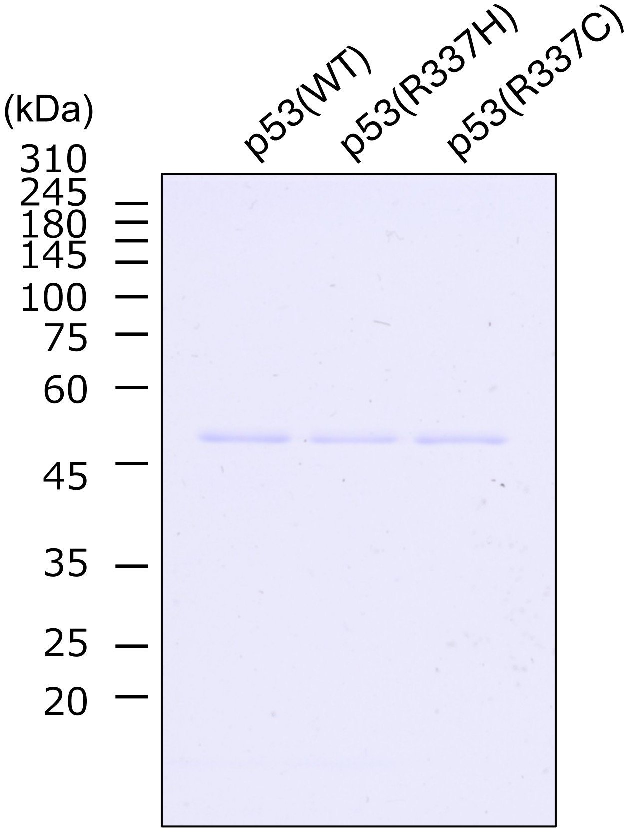

Figure S6. The SDS-PAGE analysis of purified wild-type and R337 mutant-p53 proteins. The gel was visualized by CBB-R250 staining.
